# A ureteral single-J stent in the right atrium: a case report

**DOI:** 10.1186/s13256-020-02473-y

**Published:** 2020-09-20

**Authors:** Laure Arts, Sofie Willems, Dirk Michielsen

**Affiliations:** 1 Department of Vascular Surgery, Universitair Ziekenhuis Brussel, Laarbeeklaan 101, 1090 Brussels, Belgium; 2 Department of Urology, Universitair Ziekenhuis Brussel, Laarbeeklaan 101, 1090 Brussels, Belgium

**Keywords:** J stent, intravascular migration, surgical complication, case report

## Abstract

**Background:**

J stents are commonly used to support the continuity of the urinary tract. Although intravascular, and more specific intracardiac, migrations have been described, they remain infrequent and unrecognized.

**Case report:**

We report the case of a 57-year-old Caucasian woman with an intracardial migration of a single-J stent after pelvic exenteration. The intracardiac presence of single-J stent was probably due to a perioperative misplacement of the stent in the left ovarian vein. Retrieval was done under fluoroscopic control without any adverse events.

**Conclusions:**

Intravascular migration of urological stents is uncommon but can cause serious morbidities and even mortality. Perioperative precautions must be taken to avoid this problem. In case of migration, early diagnosis and management are primordial and involve a multidisciplinary approach.

## Background

Single- and double-J stents are commonly used to maintain urinary flow from kidney to bladder, even after restoring the continuity of the urinary tract in multiple conditions. Several complications such as discomfort, urinary tract infection (UTIs), stent fracture, encrustation, and migration are well known [1]. Although intravascular [2, 3], and more specific intracardiac [2, 4], migrations of J stents have been described, these complications remain infrequent and unrecognized. We report the case of an intracardiac migrated single-J stent (SJS) in a patient with an ureterocutaneostomy that was retrieved under fluoroscopic control.

## Case presentation

We present the case of a 57-year-old Caucasian woman with a complex history of multiple abdominal surgeries involving gastric bypass, right hemicolectomy, sigmoidal perforation, and cholecystectomy. Retroperitoneal fibrosis was suspected due to previous radiotherapy for cervical carcinoma, and her abdomen was considered as hostile. Our patient was on home parenteral nutrition because of a chronic intestinal obstruction due to internal herniation.

Our patient was initially admitted because of purulent and fetid discharge from her abdominal wound. She was hemodynamically stable without fever and only a limited elevated C-reactive protein (CRP,18 mg/l) without an elevated white blood cell count (WBC, 8000/mm^3^). Several enterovesical − cutaneous and vaginal − fistulae were discovered. Because of pneumaturia, vaginal losses, sepsis with acute renal failure (hemodynamically stable with CRP level of 270 mg/l, WBC of 18,000/mm^3^, creatinine level of 5.5 mg/dl, and a SOFA [Sequential (Sepsis-related) Organ Failure Assessment] score of 4) [5] secondary to UTI and pyelonephritis, urgent bilateral nephrostomies were placed, and antibiotics were started. After recovering from sepsis, an explorative laparotomy was performed 1 month after admission. During this surgery, multiple enteric but also cutaneous, vaginal, and vesical fistulae were seen. Several small intestine resections were done, intestinal herniation restored, and loop colostomy was performed. Initially, there were good results with the retrieval of the nephrostomies. Unfortunately, replacement of the nephrostomies became necessary due to clinical deterioration with bilateral symptomatic hydronephrosis and impossibility to place double-J stents (DJS) because the ureteral origins could not be seen during cystoscopy. During the same procedure, a significant defect was observed in the posterior wall of the bladder with the development of new fistulae. We performed a total pelvic exenteration with a terminal sigmoidal colostomy. After removal of the rectum, uterus, ovaries and bladder, difficult dissection of both ureters was done by the abdominal surgeons and the left ureter was anastomosed on the right ureter by the urological team. SJS were placed in each ureter and an ureterocutaneostomy was created with the right ureter. Both SJS were visible at the skin and drained correctly.

**Fig. 1:**
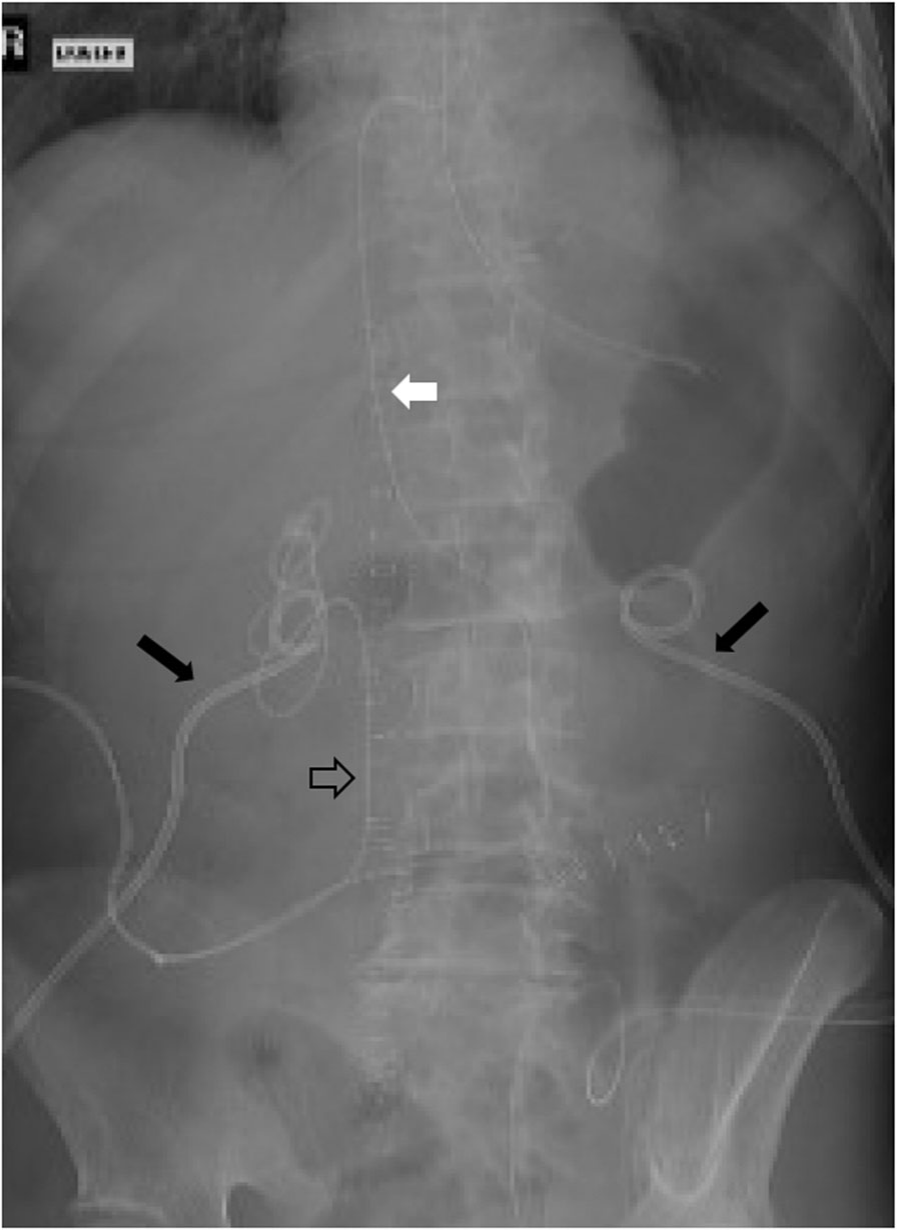
Postoperative X-ray. Black arrows: bilateral nephrostomies. White arrow: left single J stent into the right atrium. Empty arrow: right single J stent into the right kidney

Two weeks after surgery, an abdominal enhanced computed tomography (CT) scan was performed due to clinical deterioration with fecal losses through the laparotomy wound and suspicion of new enteric fistulae. On CT scan, a foreign object was seen in the right atrium. This could be followed through the inferior caval vein further to the left ureter. We could conclude that the proximal tip of the left single-J stent had migrated into the heart (Fig. 1). After examination of previous X-rays, the same position of the SJS could be seen from the very first postoperative day. Vascular and cardiac surgeons were consulted for the optimal treatment of the migrated SJS because of fear of bleeding and cardiac complications. Finally, retrieval was done by our interventional radiologist under fluoroscopic control. By placing a guidewire into the SJS at the ureterocutaneostomy and straightening the proximal tip, the SJS could be withdrawn without any complications. She was observed for 24 hours in our intensive care unit (ICU) before being transferred to the ward. Unfortunately, our patient died a month later due to several other complications and her very poor general condition.

## Discussion and conclusions

Proximal or distal migration of J stents (JS) in the urinary tract is frequent but only a few intravascular migration have been described over the past years, from the iliac vein, to the vena cava inferior to the right atrium or ventricle and even up into the pulmonary artery [2–4, 6].

Most of the time, intravascular migration is caused by perforation of the ureteral wall due to damage to fragile tissues. Associated risk factors are multiple ureteral manipulations, previous pelvic cancer, or major surgery and radiotherapy [1]. Our patient was known to have retroperitoneal fibrosis after previous radiotherapy and had also multiple urinary tract manipulations. Perforation can occur immediately by manipulation of the urinary tract when placing the JS or can be a delayed complication by erosion.

Retrograde placement of a J stent can pierce the ureter into the vascular system at the level of the adjacent intercommunicating ovarian veins or at the pyelum [4, 6]. Michalopoulos *et al.* [6] first described in 2002 the theory of migration of a DJS into the right ovarian vein up to the pulmonary artery. In 2013, Kim *et al.* [4] had a patient with DJS migration that extended from the right ovarian vein to the right atrium. Erosion of the urinary tract into the vascular system can be caused on several levels because of the adjacency of the two systems. Several publications can be found about perforation through the iliac or renal veins [3]. Those kinds of perforation could be associated with retroperitoneal bleeding, massive hematuria, abdominal or flank pain, and decreased kidney function [2, 3, 6].

Another reason for intravascular presence of a J stent, described by Kim *et al.*, [4] involves a misplacement of the stent into a transected ovarian vein that was mistaken for the ureteral end. During our complicated surgery, several surgical teams were involved. The ureteral dissection was done by abdominal surgeons and the final ureteroureterostomy by the urological team. Tissues were fibrotic due to previous radiotherapy and structures were difficult to distinguish from each other. Because of this, the left ovarian vein was probably anastomosed on the right ureter and so the SJS, placed in ‘left ureter’, was directly positioned intravascular into the right atrium without being noticed by the surgeons.

Some precautions can be taken to reduce the risk of perioperative misplacement of a ureteral stent. It is of great importance not to use any force when resistance is felt when placing the J stent. Fluoroscopic/endoscopic control during stent placement should always be used and a postoperative X-ray or ultrasound should routinely be done. Even then, misplacement could be missed because of the adjacency of the urinary and vascular systems [2, 3].

Early recognition of an intravascular, and more specific intracardial, SJS is important as it can cause severe problems such as sepsis due to UTI, pulmonary embolism, valvular disease, myocardial damage, endocarditis, or recurrent pericardial effusions. Diagnosis can be done by simple radiography, contrast-enhanced CT, venography, or cardiac ultrasound. Our patient did not present any respiratory nor cardiac complications and diagnosis was made by an accidental finding. Management of retrieval has not been clarified yet due to the scarcity of cases, however, it can be done by endoscopic, endovascular, or open surgery. Treatment is mainly dictated by the position of the distal coil of the SJS, presence of thrombus, patients’ general condition, local expertise, and available infrastructure [2, 3, 6]. Based on this, Tilborghs *et al.* [3] described, in 2019, an algorithm to choose the ideal retrieval method. We did not need this algorithm because the distal end of the SJS was still outside our patient, visible through the ureterocutaneostomy. So, retrieval was easily done by our interventional radiologist by straightening the proximal coil and gently withdrawing it without any complication.

As far as we know, this is the first description of an intracardial SJS after open positioning under direct visual control. Although we are still not certain of the exact cause of the intracardial placement of the proximal DJS tip (migration or initial misplacement), it is known that early recognition and treatment is primordial to avoid more severe complications. Perioperative precautions must be taken, and postoperative radiographs should always be done and be examined by the operators themselves.

## Data Availability

Not applicable.

## Abbreviations

CT: Computed tomography
CRP: C-reactive protein
DJS: Double-J stent
ICU: Intensive care unit
JS: J stent
SJS: Single-J stent
SOFA score: Sequential [Sepsis-related] Organ Failure Assessment score
UTI: Urinary tract infection
WBC: White blood cell count
